# Effect of far-infrared radiation therapy on von Willebrand factor in patients with chronic kidney disease

**DOI:** 10.3389/fmed.2023.1268212

**Published:** 2023-09-08

**Authors:** Cheng-Chieh Yen, Po-Chao Hsu, Chih-Ching Lin, Szu-Chia Chen, Chih-Yen Hsiao, Shang-Jyh Hwang

**Affiliations:** ^1^Division of Nephrology, Department of Internal Medicine, Ditmanson Medical Foundation Chia-Yi Christian Hospital, Chia-Yi, Taiwan; ^2^Division of Cardiology, Department of Internal Medicine, Kaohsiung Medical University Hospital, Kaohsiung Medical University, Kaohsiung, Taiwan; ^3^Faculty of Medicine, College of Medicine, Kaohsiung Medical University, Kaohsiung, Taiwan; ^4^Division of Nephrology, Department of Medicine, Taipei Veterans General Hospital, Taipei, Taiwan; ^5^School of Medicine, National Yang Ming Chiao Tung University, Taipei, Taiwan; ^6^Division of Nephrology, Department of Internal Medicine, Kaohsiung Medical University Hospital, Kaohsiung Medical University, Kaohsiung, Taiwan; ^7^Department of Internal Medicine, Kaohsiung Municipal Siaogang Hospital, Kaohsiung Medical University, Kaohsiung, Taiwan; ^8^Institute of Population Health Sciences, National Health Research Institutes, Miaoli, Taiwan

**Keywords:** a disintegrin and metalloproteinase with thrombospondin type 1 repeats 13, chronic kidney disease, far-infrared radiation, hemodialysis, von Willebrand factor

## Abstract

**Background:**

Hemostatic abnormality has contributed to vascular access thrombosis in patients with chronic kidney disease (CKD). Previous studies have demonstrated that far-infrared radiation (FIR) therapy can maintain the patency and maturity of arteriovenous fistulas of patients undergoing hemodialysis (HD). However, prolonged access bleeding is observed once FIR is conducted at the end of dialysis. FIR can block the binding of platelet and von Willebrand factor (vWF), a predictor of hemostatic abnormality and vascular access thrombosis. However, clinical studies exploring FIR and vWF are sparse.

**Methods:**

We recruited 20 HD patients, 21 CKD patients, and 20 controls to examine the alteration of vWF and a disintegrin and metalloproteinase with thrombospondin type 1 repeats 13 (ADAMTS13) following a single 40-min session of FIR therapy. In addition, the alteration of these factors in the HD group was examined following a 40-min FIR session thrice a week for 3 months.

**Results:**

A decreasing trend in the vWF activity-antigen ratio of participants in all groups following a single FIR session was observed. In addition, the ratio in the HD group was significantly lower following 3 months of FIR therapy. The subgroup analysis revealed a consistent trend and multiple regression analysis showed that participants not taking hydroxymethylglutaryl-coenzyme A reductase inhibitor, diabetes mellitus, and higher hemoglobin levels were the significant factors. The alteration of the vWF activity-antigen ratio correlated moderately to that of ADAMTS13 antigen and activity.

**Conclusion:**

FIR may alter the ratio of ultra-large vWF multimers through ADAMTS13, contributing to inhibiting platelet-endothelium interactions of CKD patients.

## Introduction

Chronic kidney disease (CKD) is defined as irreversible kidney damage or reduced kidney function with a glomerular filtration rate (GFR) of less than 60 mL/min/1.73 m^2^ that has persisted for more than 3 months (1). End-stage renal disease (ESRD) is diagnosed once the renal function of patients with CKD has declined to the extent of requiring dialysis or transplantation. Hemostatic abnormalities, a common complication of CKD, may not only lead to acute coronary heart disease or dialysis access occlusion but may also lead to gastrointestinal hemorrhage or subdural hematoma with the concurrent use of anticoagulants (2). Such problems may significantly affect the safety and quality of life of these patients and impose a huge economic burden on the healthcare system. Improving hemostatic abnormalities in patients with CKD is an important issue in clinical care.

Far-infrared radiation (FIR) is an electromagnetic wave with a wavelength between 3 and 1,000 microns (3). At present, the application of FIR in patients with CKD focused on those with ESRD, and an improvement in the maturity and patency of arteriovenous fistulas (AVFs) of HD patients has been previously reported (4–6). FIR is currently recommended as an adjuvant therapy to improve the patency of AVFs in HD patients (7). However, prolonged access bleeding occurs if the patients receive FIR therapy at the end of dialysis, raising the inference of the relationship between FIR therapy and hemostatic mechanisms.

von Willebrand factor (vWF) is a macromolecular and multimeric glycoprotein that is mainly produced by endothelial cells and platelet megakaryocytes. It is responsible for interacting with platelets to fill damaged endothelium and stabilizing the coagulation factor VIIIs to avoid its excessive consumption (8). Clinically, vWF is used not only to indicate endothelial dysfunction, but also to predict the major cardiovascular events in patients with high cardiovascular risks, prognosis of patients with ischemic stroke, portal hypertension in patients with liver cirrhosis, mortality of patients with malignancies, and AVF occlusion events in HD patients (9–16).

A recent experiment reported that FIR affects the antigen of a disintegrin and metalloproteinase with thrombospondin type 1 repeats 13 (ADAMTS13) and the ratio of the vWF multimers in human umbilical vein endothelial cells and in healthy patients (17). We hypothesized that vWF may be associated with the prolonged access bleeding after dialysis caused by FIR. Given the limited existing clinical research on this topic, this study aims to explore the effects of FIR on vWF in patients with CKD.

## Materials and methods

### Patients

This study was approved by the institutional review board of Ditmanson Medical Foundation Chia-Yi Christian Hospital (approval number: IRB2020067). Written informed consent was obtained from all participants after a comprehensive elucidation of the study objectives and procedures. The determination of a minimum sample size of 8 participants for each group was rooted in the disparities of means and standard deviations as derived from a domestic epidemiological study (18). This deliberation was carried out under the consideration of a type I error rate set at 0.05 and a type II error rate of 0.2, reflecting a judicious balance between the risk of false positives and false negatives. Subsequently, an adjustment to the sample size was executed in proportion to the available study funding. Twenty patients undergoing maintenance HD three sessions per week via an AVF with uneventful sessions for at least 3 months were recruited in this study. In addition, 21 patients with CKD who did not require dialysis and received care and health education at the nephrology outpatient clinic were recruited. A control group composed of 20 patients without any history of kidney diseases was also recruited. Patients with a history of related blood disorders, current active bleeding events, and who underwent blood transfusion therapy during the month before the study were excluded from the study. In addition, first-time users of antiplatelets or anticoagulants and patients who refused or were unable to cooperate with the study protocol were also excluded. Patient demographic data including age, blood type, smoking history, coffee consumption, AVF characteristics, comorbidities, medications, and laboratory results were collected from the electronic medical record system of the hospital and the National Health Insurance MediCloud System.

### Study design

**Figure 1:**
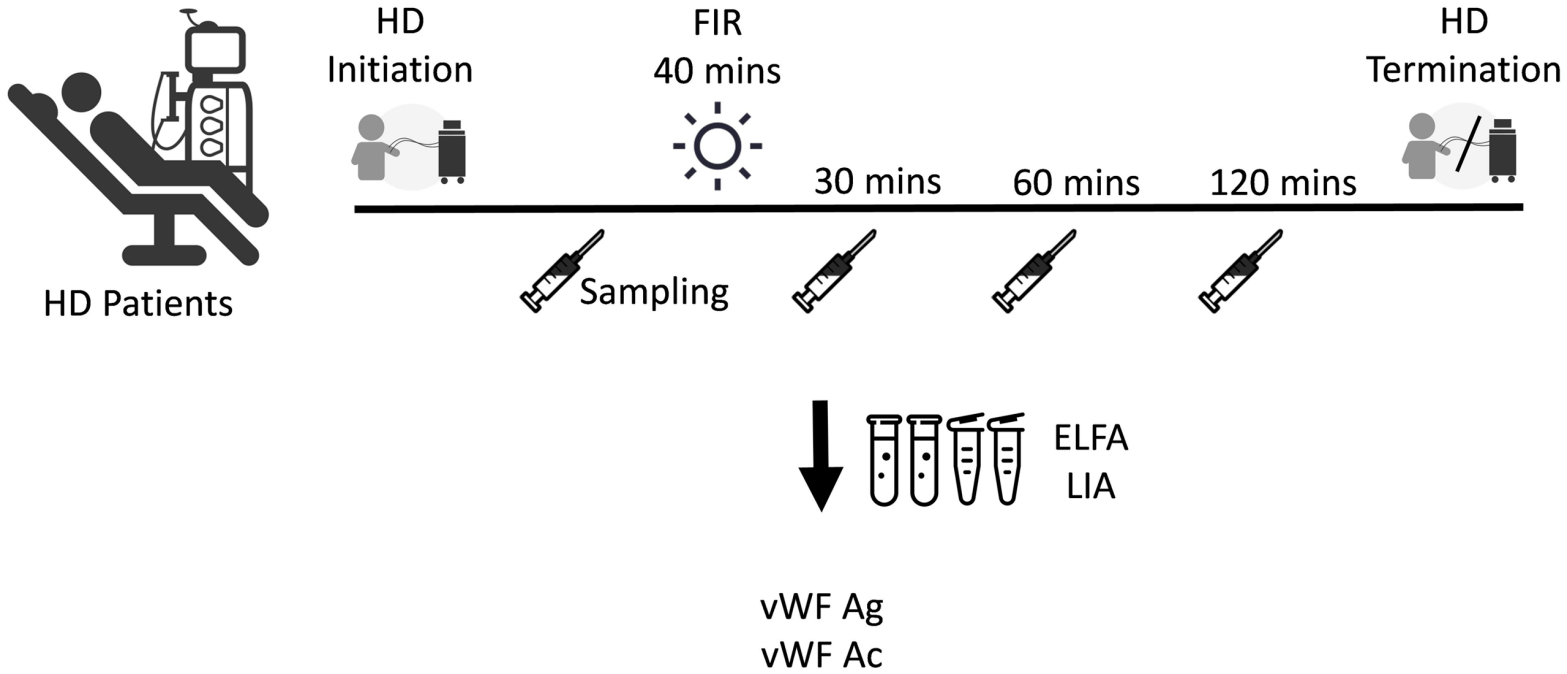
Determination of the time point for the second sampling of the antigen and activity of vWF after FIR. ELFA, enzyme-linked fluorescent assay; FIR, far-infrared radiation; HD, hemodialysis; LIA, latex immunoagglutination assay; vWF Ac, von Willebrand factor activity; vWF Ag, vWF antigen.

The relationship between vWF antigen and activity and optimal post-FIR sampling time was confirmed in 8 HD patients. The first blood sample was obtained prior to FIR. Then, the patients’ fistulas were irradiated using a FIR emitter (WS TY-101F; WS Far IR Medical Technology Co., Ltd., New Taipei City, Taiwan) for 40 min. The second, third, and fourth blood samples were obtained at 30 min, 60 min, and 120 min after FIR, respectively, to determine the optimal post-FIR sampling time to be used in the second phase of the current study (Figure 1). The vWF antigen was determined using the enzyme-linked fluorescent assay, while the vWF activity was determined using the latex immunoagglutination assay. Using these data, 30 min after FIR was determined to be the optimal time point for the second sampling during the next phase of the study.

The alteration of vWF antigen and activity and ADAMTS13 was measured in each patient group after a single FIR session. Intravenous detained needles were indwelled after confirming the position of blood vessels on the upper arm of patients with CKD and those in the control group. The first blood sample was obtained before the 40-min FIR session from the AVFs of HD patients and at the site of the detained needle indwell in the remaining patient groups. Thirty minutes after FIR, the second blood sample was obtained. The antigen and activity of ADAMTS13 were determined using an enzyme-linked immunosorbent assay.

**Figure 2:**
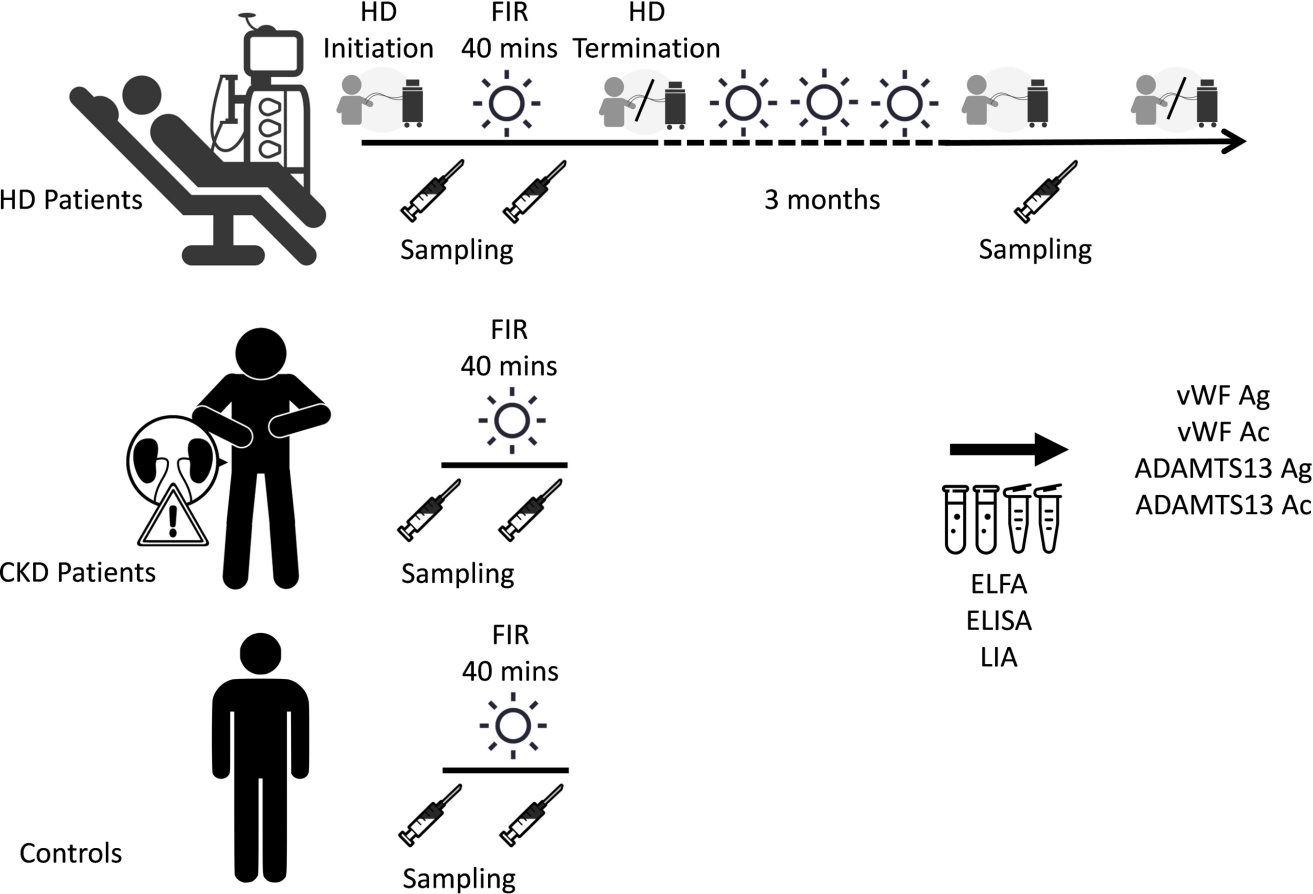
Measurements of the alteration of vWF antigen and activity and ADAMTS13 after a single session and 3 months of FIR. ADAMTS13 Ac, a disintegrin and metalloproteinase with thrombospondin type 1 repeats 13 activity; ADAMTS13 Ag, ADAMTS13 antigen; CKD, chronic kidney disease; ELFA, enzyme-linked fluorescent assay; ELISA, enzyme-linked immunosorbent assay; FIR, far-infrared radiation; HD, hemodialysis; LIA, latex immunoagglutination assay; vWF Ac, von Willebrand factor activity; vWF Ag, vWF antigen.

The third phase of the current study measured the alteration of vWF antigen and activity and ADAMTS13 in HD patients after 3 months of regular FIR therapy sessions. FIR was conducted at the site of the AVF for 40 min during each dialysis session for 3 months. Blood samples were collected after 3 months or before the first fistula complication such as abnormalities of arterial or venous pressure, difficult hemostasis after dialysis, or diminishment of the fistula thrills, regardless of whichever came first (Figure 2).

### Statistical analysis

MedCalc Statistical Software (version 20.218, MedCalc Software Ltd., Ostend, Belgium^1^) was used to conduct the statistical analyses in this study. Categorical variables were presented as frequency or percentage, while continuous variables were presented as median and quartiles. Mann–Whitney test or Wilcoxon test were used to compare the variables, when appropriate. Enter method was used for multivariate regression. The variance inflation factor was used to diagnose collinearity, which was adjusted after examining the correlation between subgroups using Spearman’s coefficient of rank correlation. Statistical significance was set at *p* < 0.05.

## Results

A total of 61 patients were recruited in this study. Patient demographic data are shown in Table 1. Of the 20 HD patients, the mean age was 60 years and 10 (50%) were males. The mean duration of dialysis was 9.5 years and the mean duration of fistula usage was 5.8 years. Of the 21 patients in the CKD group, the mean age was 71 years and 8 (38%) were males. Of the 20 patients in the control group, the mean age was 60 years and 11 (55%) were male. The prevalence of diabetes mellitus (DM) in the HD, CKD, and control groups was 40, 52, and 50%, respectively. In the HD group, the average creatinine level was 10.3 mg/dL with a corresponding estimated GFR (eGFR) of 5 mL/min/1.73 m^2^. In the CKD group, the average creatinine level was 1.6 mg/dL with a corresponding eGFR of 34 mL/min/1.73 m^2^, and the urine total protein to creatinine ratio (UPCR) was 347 mg/g. In the control group, the average creatinine level was 0.8 mg/dL with a corresponding eGFR of 91 mL/min/1.73m^2^. Fifty percent of patients in the HD group used antiplatelets, while 20% of the patients in this group used hydroxymethylglutaryl-coenzyme A (HMG-CoA) reductase inhibitors. The mean loading dose of heparin for dialysis was 750 U, and the maintenance dose was 500 U/h. One-third (33%) of the patients in the CKD group used antiplatelets, while 67% used HMG-CoA reductase inhibitors. In the control group, 45% of patients used HMG-CoA reductase inhibitors.

**Table 1:** Patient demographics.

	HD group (*N* = 20)	CKD group (*N* = 21)	Control group (*N* = 20)
Age	60 (51–68)	71 (62–73)	60 (48–66)
Male	10 (50%)	8 (38%)	11 (55%)
Type O blood	8 (40%)	8 (38%)	11 (55%)
Smokers	6 (30%)	6 (29%)	8 (40%)
Coffee consumption	5 (25%)	5 (24%)	11 (55%)
HD cause	CGN:9 (45%)		
	DN:7 (35%)		
	Others:4 (20%)		
HD vintage	9.5 (5.5–17.7)		
Fistula on the non-dominant arm	16 (80%)		
Fistula on the forearm	11 (55%)		
Fistula interventions	11 (55%)		
Fistula vintage	5.8 (3.1–11.4)		
CKD cause		DN:10 (48%)	
		CGN:5 (24%)	
		Others:6 (29%)	
Comorbidities			
Hypertension	19 (95%)	18 (86%)	11 (55%)
Cardiovascular diseases	7 (35%)	4 (19%)	1 (5%)
Heart failure	5 (25%)	4 (19%)	0 (0%)
Arrhythmia	5 (25%)	1 (5%)	2 (10%)
Cerebrovascular disease	4 (20%)	3 (14%)	1 (5%)
Peripheral vascular disease	1 (5%)	0 (0%)	0 (0%)
Diabetes mellitus	8 (40%)	11 (52%)	10 (50%)
Liver disease	8 (40%)	2 (10%)	3 (15%)
Malignant tumors	9 (45%)	1 (5%)	3 (15%)
Medication use			
Antiplatelets	10 (50%)	7 (33%)	1 (5%)
Anticoagulants	1 (5%)	2 (10%)	1 (5%)
HMG-CoA reductase inhibitors	4 (20%)	14 (67%)	9 (45%)
Loading dose of heparin (U)	750 (500–1000)		
Maintenance dose of heparin (U/h)	500 (250–500)		
Laboratory values			
Hemoglobin (g/dL)	10.7 (10.1–11.5)	11.6 (10.9–13.1)	13.9 (12.9–15.4)
Platelet count (10^3^/μL)	195 (142–214)	237 (181–275)	244 (210–310)
Prothrombin time (s)	11.1 (10.5–11.5)	10.9 (10.3–11.2)	10.9 (10.5–11.4)
International normalized ratio	1.00 (0.95–1.04)	0.94 (0.89–1.01)	0.98 (0.93–1.03)
Partial thromboplastin time (s)		31.3 (28.8–33.5)	33.8 (32.3–34.8)
Glycated hemoglobin (diabetics, %)	6.0 (5.5–7.2)	6.6 (5.8–7.5)	6.8 (6.7–7.2)
Creatinine (mg/dL)	10.3 (9.5–11.1)	1.6 (1.2–2.7)	0.8 (0.6–1.0)
Estimated glomerular filtration rate (mL/min/1.73 m^2^)	5 (4–5)	34 (19–53)	91 (80–114)
Urine protein-to-creatinine ratio (mg/g)		347 (166–1045)	

CGN, chronic glomerulonephritis; CKD, chronic kidney disease; DN, diabetic nephropathy; HD, hemodialysis; HMG-CoA, hydroxymethylglutaryl-coenzyme A.

The vWF antigen, activity, and activity-antigen ratio before and after a single FIR session are shown in Table 2. Prior to FIR, the vWF antigen and activity were significantly higher in the HD and CKD groups than in the control group. The vWF activity-antigen ratio was significantly lower in the HD group than in the control group. After a single FIR session, the characteristics of vWF antigen and activity were similar prior to FIR between groups. The alteration of vWF antigen, activity, and activity-antigen ratio after a single FIR session was not significant (Supplementary Figure S1).

**Table 2:** vWF antigen, activity, and activity-antigen ratio before and after a single session of FIR.

	HD group (*N* = 20)	CKD group (*N* = 21)	Control group (*N* = 20)
Before FIR			
vWF antigen (%)	196.1 (127.9–243.0)^**^	172.2 (132.1–273.0)^**^	126.6 (87.4–149.4)
vWF activity (%)	161.1 (104.1–177.8)^**^	154.4 (126.1–263.0)^**^	107.2 (80.0–125.4)
vWF activity-antigen ratio	0.81 (0.76–0.88)^*^	0.91 (0.81–1.00)	0.91 (0.82–0.96)
After FIR			
vWF antigen (%)	205.2 (128.3–238.8)^**^	174.5 (138.5–262.3)^**^	124.5 (87.7–151.3)
vWF activity (%)	157.9 (110.3–188.5)^**^	144.1 (118.9–249.6)^**^	105.9 (78.2–120.8)
vWF activity-antigen ratio	0.77 (0.71–0.84)^**^	0.88 (0.82–0.98)	0.87 (0.81–0.92)

*p < 0.05 compared with the control group; ^**^p < 0.01 compared with the control group. CKD, chronic kidney disease; FIR, far-infrared radiation; HD, hemodialysis; vWF, von Willebrand factor.

The vWF antigen, activity, and activity-antigen ratio before and after a single FIR session of CKD patients grouped by eGFR and UPCR are shown in Supplementary Table S1. Prior to FIR, the vWF antigen and activity were significantly higher in patients with low eGFR, while the vWF activity-antigen ratio was marginally lower among patients with low eGFR. The vWF antigen and activity were higher while the vWF activity-antigen ratio was lower as UPCR increased, although the difference between groups was not significant. After a single FIR session, the characteristics of vWF were similar prior to FIR between groups. The alteration in vWF antigen, activity, and activity-antigen ratio was not significant (Supplementary Figure S2).

**Figure 3:**
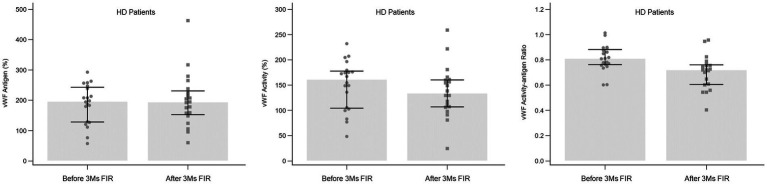
Alteration in vWF antigen, activity, and activity-antigen ratio in HD patients after 3 months of FIR. FIR, far-infrared radiation; HD, hemodialysis; vWF, von Willebrand factor.

No fistula complication was observed during the three-month period of regular FIR therapy in HD patients. The alteration in vWF antigen, activity, and activity-antigen ratio after 3 months of FIR is shown in Figure 3. The vWF activity-antigen ratio in regular FIR therapy was significantly lower than that before FIR. The subgroup analysis of the vWF activity-antigen ratio revealed a consistent decreasing trend (Table 3). The vWF activity-antigen ratio was significantly lower in patients of the male sex, with non-O blood type, who are non-smokers, who do not consume coffee, with a fistula on the non-dominant arm, with no history of fistula interventions, and with no history of cardiovascular disease, DM, liver disease, antiplatelet use, or HMG-CoA reductase inhibitor use. In addition, it was significantly lower in patients aged more than 60 years, undergoing HD for less than 9.5 years, with a loading dose of heparin less than 750 U, with a hemoglobin level of more than 10.6 g/dL, and with a platelet count of less than 195*10^3^ /μL. The multivariate regression analysis revealed that vWF activity-antigen ratio was significantly lower in patients who did not use HMG-CoA reductase inhibitors, with DM, and with higher hemoglobin levels (Table 4).

**Table 3:** Subgroup analysis of vWF activity-antigen ratio in HD patients after 3 months of FIR.

vWF activity-antigen ratio	Before FIR	After FIR	*p*
HD Patients	**0.81 (0.76–0.88)**	**0.72 (0.61–0.76)**	**<0.01**
Aged ≥60 years	0.81 (0.75–0.89)	0.67 (0.54–0.72)	0.05
Aged <60 years	0.81 (0.77–0.87)	0.75 (0.72–0.79)	0.11
Male	0.86 (0.78–0.90)	0.72 (0.60–0.76)	0.02
Female	0.77 (0.75–0.81)	0.72 (0.65–0.79)	0.19
Type O blood	0.81 (0.75–0.86)	0.76 (0.72–0.81)	0.46
Non-type O blood	0.80 (0.77–0.89)	0.67 (0.58–0.73)	<0.01
Smokers	0.84 (0.78–0.90)	0.74 (0.60–0.77)	0.16
Non-smokers	0.79 (0.76–0.87)	0.72 (0.61–0.75)	0.03
Coffee consumption	0.75 (0.60–0.78)	0.74 (0.68–0.86)	0.63
No coffee consumption	0.85 (0.78–0.90)	0.71 (0.60–0.75)	<0.01
HD vintage ≤9.5 years	0.84 (0.78–0.89)	0.73 (0.60–0.76)	0.02
HD vintage >9.5 years	0.79 (0.75–0.85)	0.72 (0.61–0.79)	0.19
Fistula on the non-dominant side	0.81 (0.76–0.87)	0.72 (0.61–0.76)	0.01
Fistula on the dominant side	0.83 (0.76–0.89)	0.70 (0.60–0.85)	0.38
Fistula on the forearm	0.78 (0.76–0.88)	0.71 (0.57–0.78)	0.07
Fistula on the upper arm	0.85 (0.76–0.88)	0.73 (0.69–0.75)	0.10
History of fistula interventions	0.78 (0.75–0.86)	0.71 (0.57–0.78)	0.24
No history of fistula interventions	0.85 (0.77–0.92)	0.72 (0.69–0.76)	0.02
Fistula vintage ≤5.8 years	0.82 (0.78–0.89)	0.72 (0.61–0.81)	0.07
Fistula vintage >5.8 years	0.77 (0.70–0.86)	0.72 (0.60–0.76)	0.10
Cardiovascular disease	0.81 (0.74–0.82)	0.79 (0.61–0.92)	0.94
No cardiovascular diseases	0.86 (0.77–0.90)	0.71 (0.61–0.73)	<0.01
Diabetes mellitus	0.87 (0.83–0.89)	0.68 (0.57–0.76)	0.02
No diabetes mellitus	0.77 (0.74–0.81)	0.72 (0.65–0.77)	0.18
Liver diseases	0.86 (0.81–0.89)	0.66 (0.55–0.77)	0.02
No liver diseases	0.77 (0.75–0.85)	0.72 (0.67–0.76)	0.15
Malignant tumors	0.78 (0.71–0.87)	0.65 (0.56–0.72)	0.10
No malignant tumors	0.81 (0.77–0.89)	0.75 (0.71–0.78)	0.05
Antiplatelet use	0.82 (0.73–0.87)	0.74 (0.72–0.77)	0.28
No antiplatelet use	0.80 (0.77–0.89)	0.67 (0.54–0.74)	0.02
HMG-CoA reductase inhibitor use	0.84 (0.79–0.88)	0.73 (0.68–0.84)	0.38
No HMG-CoA reductase inhibitor use	0.80 (0.75–0.89)	0.72 (0.58–0.76)	0.02
Loading dose of heparin ≤750 U	0.78 (0.75–0.89)	0.72 (0.55–0.75)	0.02
Loading dose of heparin >750 U	0.81 (0.78–0.88)	0.73 (0.64–0.80)	0.25
Maintenance dose of heparin ≥500 U/h	0.81 (0.78–0.87)	0.73 (0.63–0.78)	0.07
Maintenance dose of heparin <500 U/h	0.77 (0.74–0.89)	0.70 (0.56–0.74)	0.05
Hemoglobin >10.6 g/dL	0.80 (0.77–0.82)	0.72 (0.60–0.79)	0.04
Hemoglobin ≤10.6 g/dL	0.86 (0.75–0.90)	0.72 (0.65–0.76)	0.08
Platelet count >195*10^3^ /uL	0.79 (0.75–0.82)	0.76 (0.73–0.82)	0.63
Platelet count ≤195*10^3^ /uL	0.86 (0.77–0.90)	0.65 (0.56–0.72)	<0.01

The bold values revealed the overall results of the table. FIR, far-infrared radiation; HD, hemodialysis; HMG-CoA, hydroxymethylglutaryl-coenzyme A; vWF, von Willebrand factor.

**Table 4:** Multivariate regression analysis of vWF activity-antigen ratio in HD patients after 3 months of FIR.

Variable	β	SE	*p*	VIF
Constant	0.77			
Age	<−0.01	<0.01	0.12	2.78
Blood type	−0.06	0.04	0.18	2.36
Smokers	0.06	0.05	0.26	2.68
Coffee consumption	0.06	0.04	0.23	2.02
Fistula interventions	0.11	0.05	0.07	3.28
Cardiovascular diseases	0.08	0.03	0.06	1.53
Diabetes mellitus	−0.20	0.05	<0.01	3.21
Liver diseases	−0.08	0.04	0.10	2.11
Antiplatelet use	0.08	0.03	0.05	1.51
HMG-CoA reductase inhibitor use	0.21	0.06	0.01	3.34
Loading dose of heparin	<0.01	<0.01	0.08	4.11
Hemoglobin	−0.07	0.02	<0.01	2.74
Platelet count	<0.01	<0.01	0.75	3.51

HD, hemodialysis; FIR, far-infrared radiation; HMG-CoA, hydroxymethylglutaryl-coenzyme A; SE, standard error; VIF, variance inflation factor.

**Figure 4:**
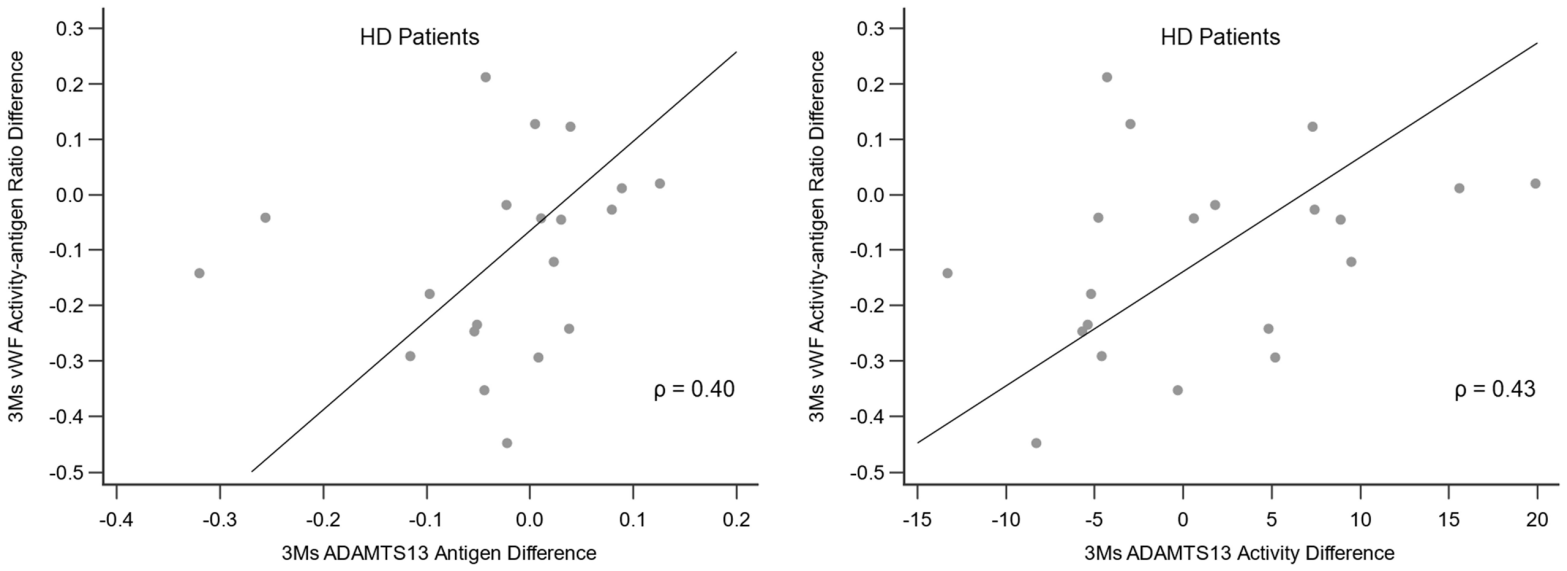
Correlation between the alteration in vWF activity-antigen ratio and the alteration in ADAMTS13 antigen and activity in HD patients after 3 months of FIR. ADAMTS13, a disintegrin and metalloproteinase with thrombospondin type 1 repeats 13; FIR, far-infrared radiation; HD, hemodialysis; vWF, von Willebrand factor.

The association between the alteration in the vWF activity-antigen ratio and the alteration in ADAMTS13 antigen and activity in HD patients after 3 months of FIR therapy is shown in Figure 4. A moderate positive correlation was observed between the alteration of the vWF activity-antigen ratio and the alteration of ADAMTS13 antigen and activity.

## Discussion

FIR therapy at the end of HD leads to prolonged hemostasis. This study observed that 3 months of FIR therapy significantly reduced vWF activity-antigen ratio in HD patients, positively correlating with changes in ADAMTS13 antigen and activity. These findings support the link between FIR therapy and hemostatic mechanisms.

The release of vWF can be stimulated or unstimulated. Unstimulated release involves polymers of different molecular weights stably released from endothelial cells, including ultra-large vWF multimers (ULvWFs), large vWF multimers, medium vWF multimers, low vWF multimers, and vWF dimers. The stimulated release from the Weibel-Palade bodies of endothelial cells is triggered by thrombin, fibrin, or collage, thus releasing ULvWFs and large vWF multimers. As ULvWFs and large vWF multimers can lead to vascular obstruction, the metalloproteinase ADAMTS13, which is mainly synthesized by hepatic stellate cells, binds to the A2 domain of vWF, cutting the molecules to reduce the molecular weight of vWF multimers (19).

The vWF activity measurement involves detecting the binding of vWF and the glycoprotein 1b of platelets. ULvWF contains more A1 domains that can bind to platelet glycoprotein 1b than other multimers. Therefore, increased ULvWF levels is associated with higher vWF activity. The vWF activity is considered as the quantity of ULvWF, while the vWF activity-concentration ratio is the ratio of ULvWF to all vWF.

Patients with CKD had elevated vWF antigen levels than patients in the control group due to endothelial cell dysfunction caused by the accumulation of uremic toxins (20). A prospective observational study conducted by Huang et al. reported that the vWF activity of CKD patients was higher than that of patients in the control group and was inversely proportional to the GFR of patients (21). In addition, Warrell et al. reported that the vWF antigen and activity were higher in HD patients than in the control group with normal renal function (22). Gralnick et al. reported that there was elevated plasma vWF antigen and activity but reduced vWF activity-antigen ratio in HD patients compared to patients with normal renal function. Decreased ULvWF ratio was observed in the gel electrophoresis of vWF (23). Marchini et al. also recently reported similar findings as that of Gralnick et al. (24). The results of the baseline vWF characteristics in this study are consistent with those of previous studies.

To improve the quality of medical care and delay ESRD-associated complications for CKD patients in Taiwan (25), the Health Promotion Administration, the National Health Insurance Administration of the Ministry of Health and Welfare, and the Taiwan Society of Nephrology jointly promoted a care and health education program for pre-ESRD patients. Proposed in 2003, the program targeted CKD patients with an eGFR of less than 45 mL/min/1.73 m^2^ who did not require dialysis. A patient-centered holistic care plan was implemented in outpatient clinics (26) and resulted in decreased incidences of dialysis, mortality, and medical insurance expenditures (27). In addition, proteinuria is an important predictor of prognosis in CKD patients. A higher concentration of proteinuria is associated with a higher probability of decreased renal function, ESRD, and mortality (28, 29). In this study, CKD patients were divided into groups based on eGFR of 45 mL/min/1.73 m^2^ and UPCR to compare the alteration in vWF antigen and activity. The results of baseline vWF characteristics in association with eGFR and UPCR were consistent with those of previous studies (20, 21, 30, 31).

This study revealed that the vWF activity-antigen ratio in patients undergoing HD decreased significantly after 3 months of FIR sessions. Patient blood type, age, sex, smoking status, coffee consumption, comorbidities (such as DM and liver disease), and use of HMG-CoA reductase inhibitor or heparin usage can also affect vWF characteristics (32–39). The use of antiplatelet medications, erythrocytes, and platelets are important factors for primary hemostasis (40). Therefore, we included factors affecting vWF characteristics in the subgroup analysis. We observed a consistent alteration of the vWF activity-antigen ratio in all subgroups, which may be associated with the clinical observation that delayed FIR leads to prolonged hemostasis in all HD patients.

Multivariate regression analysis revealed that the vWF activity-antigen ratio was significantly reduced in those who did not use HMG-CoA reductase inhibitors, with DM, and with higher hemoglobin levels. HMG-CoA reductase inhibitors were observed to decrease vWF antigen levels and improve endothelial function, though Özkurt et al. reported that unchanged vWF antigen levels after 6 months of atorvastatin administration in patients undergoing HD and peritoneal dialysis (41). Hyperglycemia may lead to abnormal endothelial function in patients with DM, resulting in increased vWF antigen and activity. Stehouwer et al. reported that the vWF antigen significantly increased with microalbuminuria (42), and Domingueti et al. reported that the vWF antigen levels increased as renal function decreased and albuminuria increased in type 1 DM patients (43). Type 2 DM patients with nephropathy had higher vWF antigen levels than those with type 2 DM without albuminuria, impaired glucose tolerance, and a type 2 DM family history (44). Erythrocytes can stabilize activated vWFs by binding the platelet glycoprotein 1bs. In addition, they compete with ADAMTS13s to bind with vWFs, thereby reducing the cutting of vWF decomposition. Patel et al. reported increased vWF antigen and activity in patients with non-dialysis CKD and anemia (45). No studies regarding the effects of HMG-CoA reductase inhibitor use, DM, and hemoglobin on the vWF activity-antigen ratio in patients with CKD have been reported. Patients who do not use HMG-CoA reductase inhibitors, with DM, and with higher hemoglobin levels are supposed to be prothrombotic with higher vWF characteristics. The results of multivariate regression analysis in this study revealed that FIR therapy may be more beneficial among those patients.

Hwang et al. reported that ADAMTS13 mRNA, ADAMTS13 antigen, and vWF D4-CK domain antigen of human umbilical vein endothelial cells increased after 30 min of FIR, resulting in a decreased platelet binding capacity. An electrophoretic analysis of vWF polymers revealed that the ratio of ULvWF decreased after FIR. In addition, increased ADAMTS13 antigen levels and decreased ULvWF ratios were also observed in healthy patients after FIR. Therefore, FIR therapy may result in increased ADAMTS13 levels that can cut vWF into smaller multimers to reduce platelet binding, ultimately inhibiting platelet aggregation (17). The alteration in the vWF activity-antigen ratio observed in our study after FIR therapy matched those seen in the previous study on the ULvWF ratio. However, the alteration in ADAMTS13 antigen and activity observed in our study differed from those reported in the previous study. Future studies including vWF electrophoresis, platelet aggregation analysis, and measurements of factor VIII alteration are warranted to verify and determine mechanisms.

FIR therapy has maintained the AVF patency of HD patients by inhibiting vascular endothelial inflammation (4, 5, 46). This study observed that FIR significantly reduced vWF activity-antigen ratio in HD patients, especially in prothrombotic subgroups. We theorized that FIR may inhibit platelet-endothelium interactions by altering the proportion of ULvWFs in HD patients through ADAMTS13, providing another perspective on the protective effect of FIR on AVFs.

This study has several limitations. This purposive-sampling study population was small in number to adjust the differences between the groups. However, we focused on the alteration in vWF characteristics before and after FIR therapy and analyzed the differences in patient demographics by subgroup analysis. In addition, alteration in the concentration of uremic toxins, heparinization, and ultrafiltration that occurred between the sampling periods may affect vWF characteristics. Moreover, all blood samples were obtained from the same site, therefore, distinguishing the local and systemic effects on vWF cannot be conducted. Given the uneventful 3-month observation period and absence of complications in the AVFs, it is plausible that the considerable flow volume and brisk flow rate in AVFs played a role. The impact of FIR on vWF properties might have extended beyond the local level and involved systemic circulation. Lastly, vWF was measured before and after a single FIR session in the CKD and control groups. The effects of 3 months of FIR therapy cannot be determined in these patients.

In conclusion, a single FIR session reduced the vWF activity-antigen ratio in all patients. Moreover, 3 months of FIR therapy also significantly reduced the ratio in HD patients, the alteration of which was positively correlated with that of ADAMTS13 antigen and activity. Therefore, FIR therapy may have altered the proportion of ULvWF through ADAMTS13 in CKD patients, ultimately resulting in prolonged hemostasis. In addition to the improvement of vascular endothelial dysfunction, FIR may also maintain AVF patency by inhibiting platelet-endothelium interactions.

## Data availability statement

The original contributions presented in the study are included in the article/Supplementary material, further inquiries can be directed to the corresponding author.

## Ethics statement

The studies involving humans were approved by Ditmanson Medical Foundation Chia-Yi Christian Hospital (approval number: IRB2020067). The studies were conducted in accordance with the local legislation and institutional requirements. The participants provided their written informed consent to participate in this study.

## Author contributions

C-CY: Conceptualization, Data curation, Formal analysis, Funding acquisition, Investigation, Methodology, Project administration, Resources, Software, Validation, Visualization, Writing – original draft. P-CH: Visualization, Writing – review & editing. C-CL: Supervision, Validation, Writing – review & editing. S-CC: Methodology, Writing – review & editing. C-YH: Funding acquisition, Resources, Writing – review & editing. S-JH: Conceptualization, Funding acquisition, Methodology, Supervision, Writing – review & editing.

## Funding

The author(s) declare financial support was received for the research, authorship, and/or publication of this article. This study was funded by the Ditmanson Medical Foundation Chia-Yi Christian Hospital Research Program (R110-003).

## Conflict of interest

The authors declare that the research was conducted in the absence of any commercial or financial relationships that could be construed as a potential conflict of interest.

## Publisher’s note

All claims expressed in this article are solely those of the authors and do not necessarily represent those of their affiliated organizations, or those of the publisher, the editors and the reviewers. Any product that may be evaluated in this article, or claim that may be made by its manufacturer, is not guaranteed or endorsed by the publisher.

## Footnotes

^1^https://www.medcalc.org; 2023.
