# Supplementary material for: Effect of far-infrared radiation therapy on von Willebrand factor in patients with chronic kidney disease
Source: Front Med (Lausanne). 2023 Sep 8;10:1268212. doi: 10.3389/fmed.2023.1268212 (PMC10514495; doi:10.3389/fmed.2023.1268212)
Supplement: Supplementary file 1 [file Data_Sheet_1.docx]

**Supplementary Material**


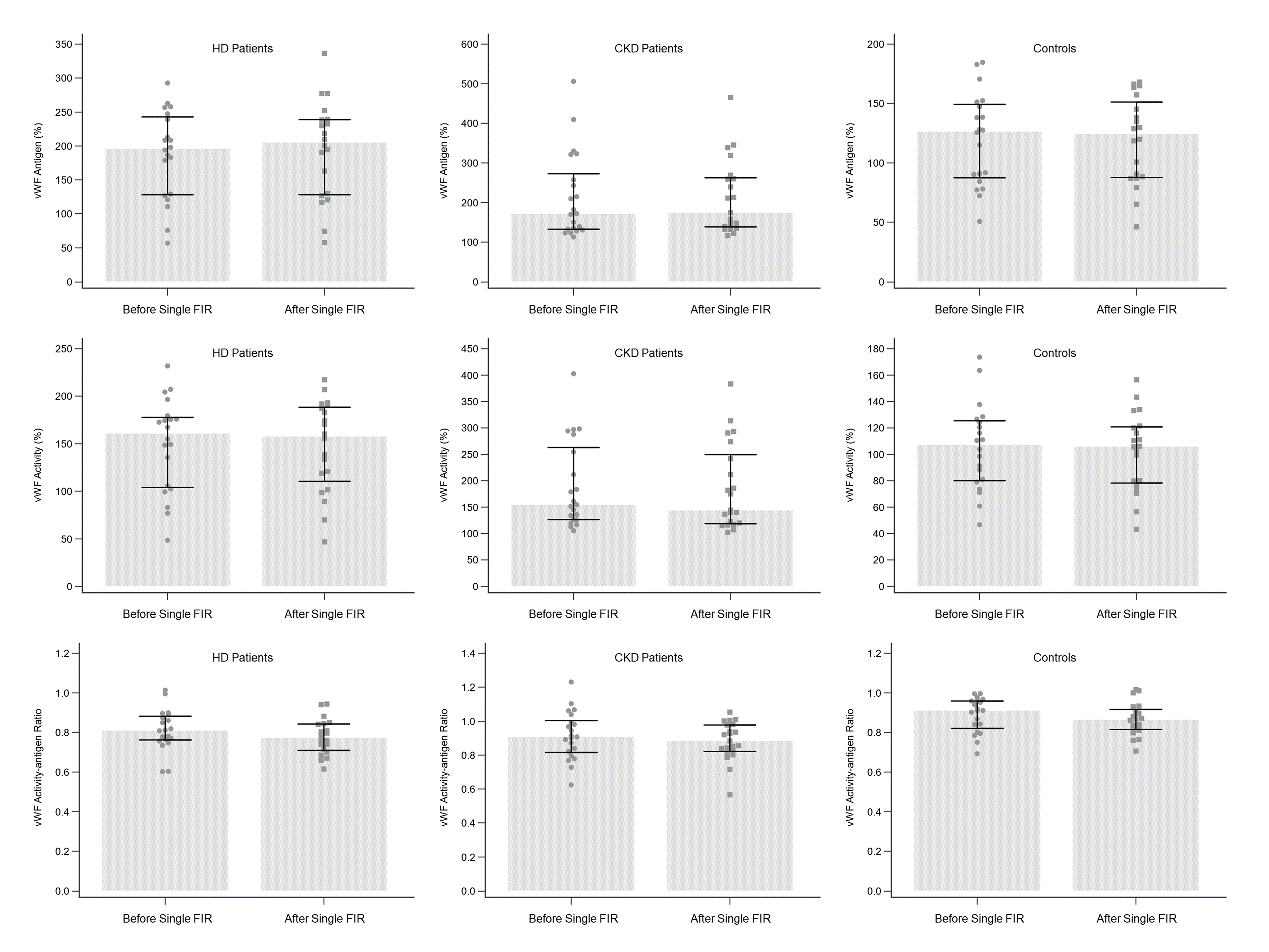


Supplementary Figure 1. Alteration in vWF antigen, activity, and activity-antigen ratio after single session of FIR.

Abbreviations: CKD: chronic kidney disease; FIR: far-infrared radiation; HD: hemodialysis; vWF: von Willebrand factor


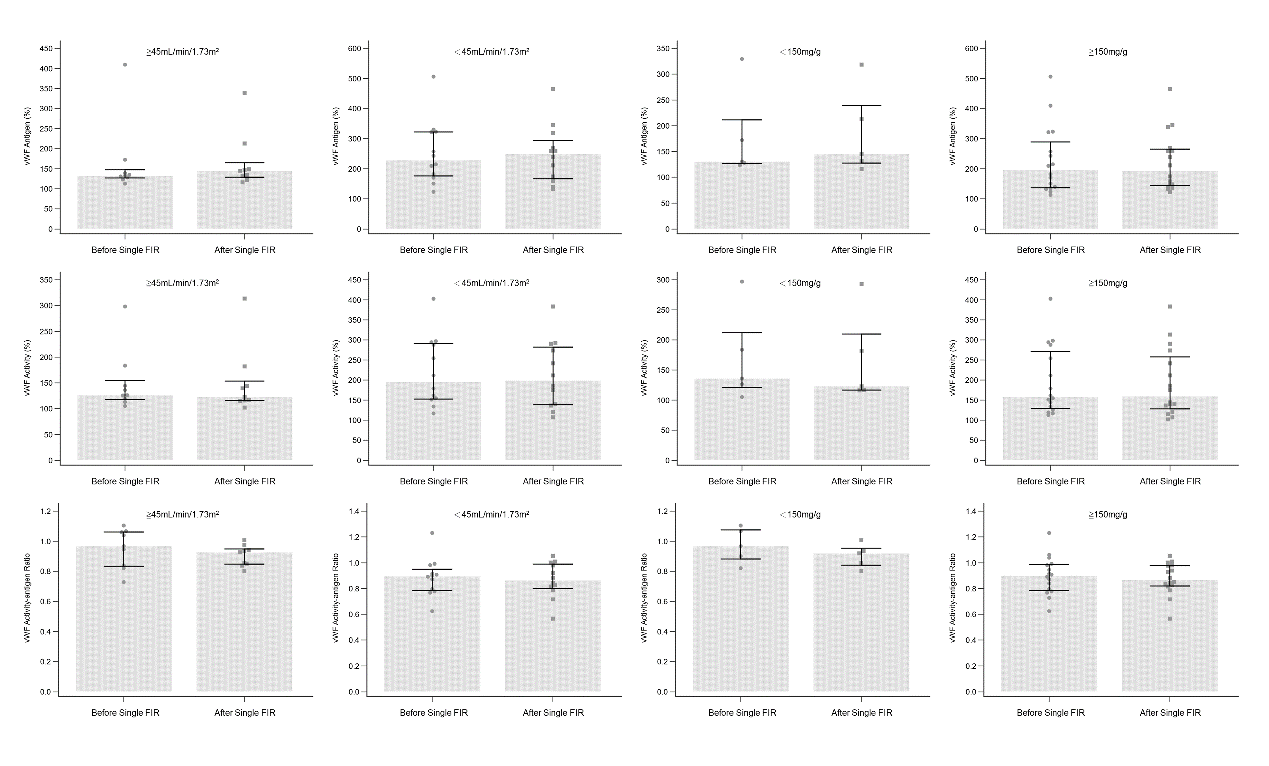


Supplementary Figure 2. Alteration in vWF antigen, activity, and activity-antigen ratio after single session of FIR in CKD patients grouped by eGFR and UPCR.

Abbreviations: CKD: chronic kidney disease; eGFR: estimated glomerular filtration rate; FIR: far-infrared radiation; UPCR: urine total protein to creatinine ratio; vWF: von Willebrand factor

Supplementary table 1. vWF antigen, activity, and activity-antigen ratio in CKD patients before and after a single FIR session

| eGFR | ≧45mL/min/1.73m^2^  (N = 9) | ＜45mL/min/1.73m^2^  (N = 12) | *p* |
| --- | --- | --- | --- |
| **Before single FIR session** |  |  |  |
| vWF antigen (%) | 132.6 (126.8 - 147.5) | 228.6 (176.2 - 321.9) | 0.02 |
| vWF activity (%) | 126.3 (117.8 - 154.4) | 195.3 (152.8 - 290.9) | 0.03 |
| vWF activity-antigen ratio | 0.97 (0.83 - 1.06) | 0.90 (0.79 - 0.95) | 0.23 |
| **After single FIR session** |  |  |  |
| vWF antigen (%) | 144.9 (129.1 - 164.8) | 249.2 (166.3 - 293.9) | 0.03 |
| vWF activity (%) | 122.9 (116.0 - 153.6) | 198.6 (138.2 - 282.0) | 0.08 |
| vWF activity-antigen ratio | 0.93 (0.85 - 0.95) | 0.86 (0.80 - 0.99) | 0.57 |
| UPCR | ＜150 mg/g  (N = 5) | ≧150 mg/g  (N = 16) | *p* |
| **Before single FIR session** |  |  |  |
| vWF antigen (%) | 130.6 (126.8 - 211.6) | 195.7 (137.0 - 289.0) | 0.28 |
| vWF activity (%) | 136.1 (121.0 – 212.0) | 157.5 (129.7 - 271.3) | 0.56 |
| vWF activity-antigen ratio | 0.97 (0.88 - 1.08) | 0.90 (0.79 - 0.99) | 0.25 |
| **After single FIR session** |  |  |  |
| vWF antigen (%) | 144.9 (127.6 - 239.4) | 192.9 (143.7 - 264.6) | 0.30 |
| vWF activity (%) | 122.9 (116.6 - 209.7) | 159.4 (128.2 - 257.6) | 0.56 |
| vWF activity-antigen ratio | 0.92 (0.84 - 0.95) | 0.87 (0.82 - 0.98) | 0.74 |

Abbreviations: CKD: chronic kidney disease; eGFR: estimated glomerular filtration rate; FIR: far-infrared radiation; UPCR: urine total protein to creatinine ratio; vWF: von Willebrand factor
